# Matrix metalloproteinases (MMPs) mediate leukocyte recruitment during the inflammatory phase of zebrafish heart regeneration

**DOI:** 10.1038/s41598-018-25490-w

**Published:** 2018-05-08

**Authors:** Shisan Xu, Sarah E. Webb, Terrence Chi Kong Lau, Shuk Han Cheng

**Affiliations:** 1 Department of Biomedical Sciences, College of Veterinary Medicine and Life Science, City University of Hong Kong, Kowloon Tong, Kowloon, Hong Kong SAR PR China; 2 Division of Life Science, The Hong Kong University of Science and Technology, Clear Water Bay, Kowloon, Hong Kong SAR PR China; 3 State Key Laboratory of Marine Pollution (SKLMP) at City University of Hong Kong, Kowloon Tong, Kowloon, Hong Kong SAR PR China; 4 Department of Materials Science and Engineering, College of Science and Engineering, City University of Hong Kong, Kowloon Tong, Kowloon, Hong Kong SAR PR China

## Abstract

In zebrafish, the role of matrix metalloproteinases (MMPs) in the inflammatory phase of heart regeneration following cryoinjury remains poorly understood. Here, we demonstrated an increase in MMP enzymatic activity and elevated expression of *mmp9* and *mmp13* in the injured area (IA) of hearts from as early as 1 day post-cryoinjury (dpc). Treatment with the broad-spectrum MMP inhibitor, GM6001, during the first week after cryoinjury resulted in impaired heart regeneration, as indicated by the larger scar and reduced numbers of proliferating cardiomyocytes. GM6001 also significantly reduced the number of leukocytes to the IA at 0.5 dpc to 4 dpc. Specific inhibition of both MMP-9 and MMP-13 also resulted in impaired regeneration and leukocyte recruitment. However, chemokine rescue with recombinant CXCL8 and CCL2 restored the recruitment of macrophages and the cardiac regenerative capability in GM6001-treated fish. MMP-9 and MMP-13 cleaved zebrafish CXCL8 at the same site, and the truncated form was more chemotactic than the intact form. In contrast, CCL2 did not have an MMP-9 or MMP-13 cleavage site. Together, these data suggest that MMPs might play a key role in the inflammatory phase of heart regeneration in zebrafish, by mediating leukocyte recruitment via the activation of chemokines.

## Introduction

Unlike adult mammals, adult zebrafish exhibit an exceptional regenerative capability to replace damaged ventricular tissues with new cardiac muscle cells, and they achieve robust anatomical and functional recovery. In 2002, Poss and Keating were the first to report the remarkable level of heart regeneration in zebrafish following ventricular amputation^1^. Since then, additional injury models such as genetic ablation and cryoinjury have been developed^2–6^, and many of the associated cellular and molecular events that take place during regeneration have been dissected by various groups^7^. One of the major cellular events that is known to occur involves cardiomyocytes in the damaged myocardium, which proliferate, migrate and display unique dedifferentiated features^8–11^. Epicardial-derived cells migrate to the injured myocardium where they give rise to perivascular cells and myofibroblasts, and epicardial regeneration is regulated by PDGF and Hedgehog signaling^12–15^. Biomechanical tension affects the cell-cycle dynamics and creates distinct subpopulations of epicardial cells during regeneration^16^. Regeneration of the endocardium involves Notch signaling and *serpine1* expression, and these regulate the proliferation and dedifferentiation of cardiomyocytes; up-regulate the expression of inflammatory genes; and recruit leukocytes to the site of injury^17^. Likewise, nerves guide myocyte proliferation and regulate immune gene expression in the regenerating zebrafish heart, whereas telomerase activation is required for cardiomyocyte proliferation and fibrotic tissue regression^18,19^.

In the zebrafish heart following cryoinjury there is localized tissue damage in the ventricle, and the resulting scar resembles the damage caused by a myocardial infarct in adult mammals. However, one major difference between adult mammals and zebrafish is that in the former, the fibrotic scar that forms remains irreversible, whereas in the latter, the scar is replaced by functional myocardium. Indeed, the cellular events involved in scar formation and resolution have been studied extensively in zebrafish^4,15,20^. For example, it is known that elevated collagenolytic activity starts 14 dpc, but by 30 dpc the level of activity is much reduced. In contrast, the expression level of various MMP transcripts are known to peak at 3 dpc, but they have declined by 7 dpc, and have returned to basal levels by 14 dpc^20^. It was thus suggested that type-I collagenases might play a role during the early phases of injury, namely the inflammation and scar formation periods. Together, these previous studies indicated that MMPs are most active from ~14 dpc to ~60 dpc to promote scar degradation. However, as MMPs are also known to cleave cytokines^21^, their role(s) in the inflammatory response, which starts shortly after cryoinjury, are still to be determined.

Inflammation is the major early response to injury, and in zebrafish this occurs during the first 7 dpc and consists of both acute inflammation and resolution events. Histological examination and electron microscopy have revealed the infiltration of leukocytes into a wound at 1, 3 and 7 dpc^6^. In addition, using immunohistochemistry, the dynamics of leukocyte infiltration have been demonstrated at 6 hours post cryoinjury (hpc), 2 dpc and 7 dpc^22^. It was shown that both macrophages and neutrophils were detected at 6 hpc, but there were more neutrophils than macrophages at this time point. The number of neutrophils peaked at 2 dpc but they declined thereafter such that by 7 dpc there were fewer cells than were observed at 6 hpc. In contrast, the number of macrophages increased between 6 hpc and 7 dpc, indicating that these cells are continually recruited during the first 7 days following cryoinjury^22^. Similar changes in the numbers of macrophages and neutrophils have also been demonstrated following the genetic ablation of cardiomyocytes in the heart. Increased numbers of macrophages were demonstrated by the prominent expression of *pu.1* at 3 days post-injury (dpi) and 5 dpi^23^. In addition, ventricular resection experiments using the Tg(*coro1a*:EGFP) line of zebrafish, where leukocytes are labeled with EGFP, demonstrated that these cells first appeared at ~3 days post amputation (dpa), and the numbers peaked at 7 dpa and 14 dpa, after which they gradually disappeared at 19 dpa^24^. While these different studies demonstrate a broad inflammatory response occurring during the first 7 days in different injury models, the dynamics of recruitment, dispersal and peak densities of the neutrophil and macrophage populations need further exploration. In addition, the chemokines CXCL8 and CCL2 have been shown to play key roles in leukocyte migration^25,26^; however, their specific roles and modifications during heart regeneration in zebrafish are still largely unknown.

We hypothesize that as zebrafish heart regeneration is a highly orchestrated and tightly controlled process, MMPs play key roles in the inflammatory response during the first 7 days after cryoinjury. In this study, therefore, we characterized the activity and expression of MMPs and explored their critical roles in the inflammatory phase using both broad spectrum and specific inhibitors. We also investigated whether MMPs might play an essential role in the recruitment of leukocytes, as well as their impact upon the regenerative capacity and cleavage of chemokines. We discuss the subtle differences that occur in the MMP cleavage of CCL2, which might help to explain the divergent outcomes following ventricular damage in zebrafish and mammals.

## Results

### Increase in both MMP enzymatic activity and mmp expression in the injured area after cryoinjury

**Figure 1:**
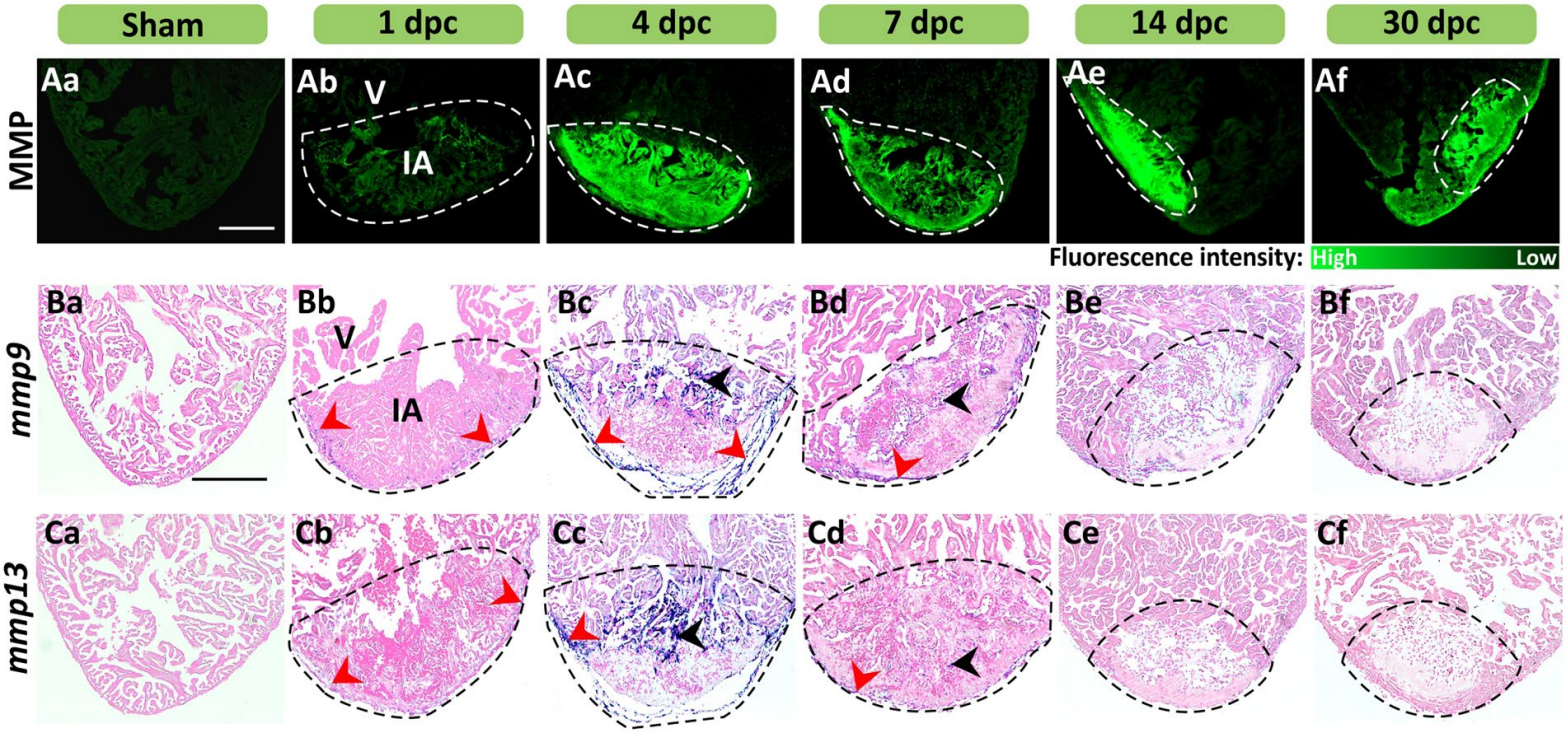
MMP activity and *mmp9* and *mmp13* expression are enhanced at sites of injury in the heart. (**A**) *In situ* zymography was conducted to show the activity of MMP (in green) in representative cryosections of (Aa) intact hearts and in (Ab–Af) injured hearts at 1–30 dpc. (**B**,**C**) *In situ* hybridization was conducted to show the expression of (**B**) *mmp9*, and (**C**) *mmp13* (blue labeling), in representative paraffin sections of (Ba, Ca) intact and (Bb–Bf, Cb–Cf) cryoinjured ventricles at 1–30 dpc. The sections were counterstained with Fast Red (pink labeling) to label all the cells. The red and black arrowheads indicate regions of *mmp* expression in the epicardium and myocardium, respectively. In each panel, the regions bounded by the dashed lines indicate the injured area (IA) of the ventricle (V). Scale bars: 200 µm.

To investigate the roles of MMP during the acute inflammation, resolution and tissue remodeling phases of regeneration, we profiled the level of MMP enzymatic activity in the IA using *in situ* zymography (Fig. 1A). Following cryoinjury, a relatively low level of MMP activity was detected at 1 dpc but it increased considerably at 4 dpc and it was still elevated at 30 dpc. The expression profiles of *mmp9* and *mmp13* (genes that encode a gelatinase and a collagenase, respectively) were also investigated from 1 dpc to 30 dpc as well as intact heart via *in situ* hybridization on paraffin sections (Fig. 1B and C). At 1 dpc, low levels of *mmp9* and *mmp13* expression were mainly detected in the epicardial layer of the IA (see red arrowheads in (Fig. 1Bb,Cb). By 4 dpc, the level of expression of both *mmp9* and *mmp13* had increased considerably and, in addition to the epicardial layer (see red arrowheads in (Fig. 1Bc,Cc), the expression expanded deep within the IA (see black arrowheads in (Fig. 1Bc,Cc). By 7 dpc, the level of expression and pattern of *mmp9* and *mmp13* returned to levels equivalent to those at 1 dpc, and at 14 dpc and 30 dpc, the level of expression of both genes was very low. These data suggest that MMPs were activated at 1 dpc, prior to the up-regulated expression of *mmp* genes at 4 dpc.

Vimentin-positive fibroblasts are associated with collagen XII-labeled fibers, and are reported to contribute to the structural framework surrounding the epicardium from 7 dpc onward^4,15^. Here, we showed via immunohistochemistry, a detectable but low level of vimentin-positive labeling at 1 dpc (Fig. S1Ab). The level of vimentin then increased considerably in both the interior of the wound and the surrounding epicardial wall at 4 dpc (Fig. S1Ac) and it was still at this elevated level and pattern of expression at 7 dpc (Fig. S1Ad). A combination of FISH (of *mmp9* and *mmp13*), and immunohistochemistry (of vimentin) on paraffin sections showed that there was ~66% over-lap of the vimentin/*mmp* gene data at 4 dpc (Fig. S1B). These results suggest that fibroblasts might secrete MMPs during the inflammatory phase following cryoinjury.

### GM6001 inhibits heart regeneration

**Figure 2:**
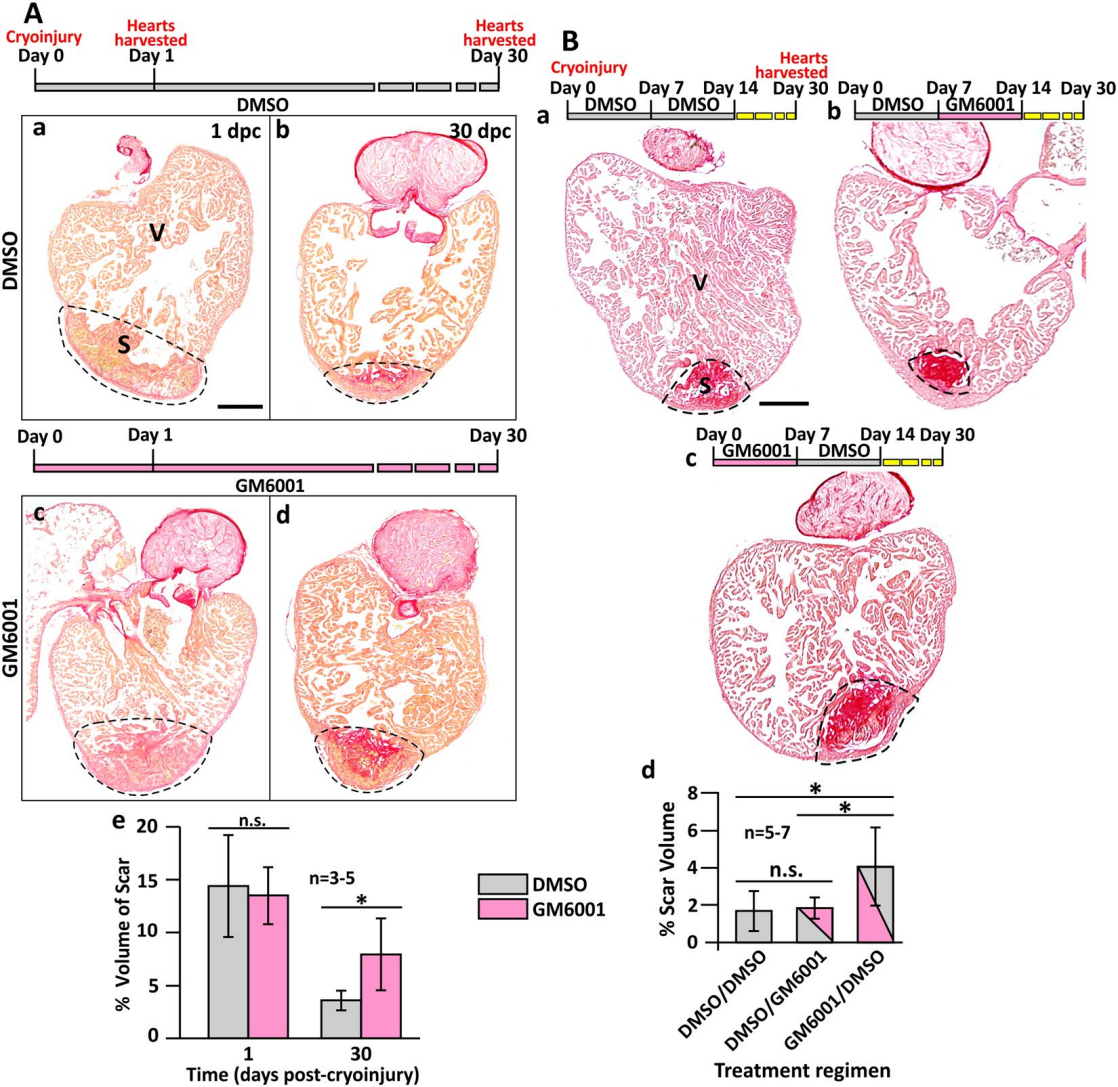
GM6001 inhibits heart regeneration after cryoinjury. (**A**) Representative picrosirius red stained paraffin sections of wild type fish hearts at (Aa, Ac) 1 dpc and (Ab, Ad) 30 dpc, after the fish were injected with either DMSO (controls) or GM6001 (prepared in DMSO). ‘V’ and ‘S’ are the ventricle and scar (of the injured area), respectively. Scale bar: 200 µm. (Ae) Bar graph to show the percentage of the scar volume in the DMSO- and GM6001-injected fish at 1 dpc and 30 dpc. The data are expressed as mean ± standard deviation of n = 3 to 5 hearts. The asterisk indicates GM6001 data that are significantly different from the DMSO controls at p < 0.05, two-tailed t-test. (**B**) Representative picrosirius red stained paraffin sections of wild type hearts harvested at 30 dpc, after the fish were injected with: (Ba) DMSO every day for the first 14 dpc; (Bb) DMSO for the first 7 dpc and GM6001 for the next 7 dpc; or (Bc) GM6001 for the first 7 dpc and DMSO for the next 7 dpc. ‘V’ and ‘S’ are the ventricle and scar (of the injured region), respectively. Scale bar: 200 µm. (Bd) Bar graph to show the percentage of scar volume in the ventricle following the different DMSO and GM6001 injection regimens. The data are expressed as mean ± standard deviation of n = 5 to 7 hearts. The asterisks indicate significantly different data at p < 0.05, and ‘n.s.’ indicates results where no statistically significant differences were found, one-way ANOVA with LSD Post-hoc test.

To investigate if MMPs might play a role in heart regeneration, zebrafish were injected once a day intraperitoneally with either GM6001 (a pan-matrix metalloproteinase inhibitor) or DMSO (as the vehicle control), and the volume of the IA (scar) at 1 dpc and 30 dpc was then visualized via picrosirius red staining of paraffin sections (Fig. 2Aa–Ad). The percentage volume of the collagen-rich scar was calculated relative to the volume of the ventricle (Fig. 2Ae). At 1 dpc, the scar volumes in the control and treated groups did not differ significantly, with values of ~14.4% and ~13.5% being calculated, respectively (*p* = 0.8). However, at 30 dpc, the percentage of the scar volume of the control group was ~3.7%, which was significantly smaller than that of the GM6001-injected group, which was ~8.0% (*p* = 0.038, Fig. 2Ae). These results indicate that the resolution of the scar and its replacement with myocardium was impaired when MMP activity was inhibited during the inflammatory-, scar formation- and scar degradation-phases of regeneration. In order to determine which of these 3 phases might be most sensitive to MMP activity, zebrafish were: (a) Injected with DMSO for 14 days as a control; (b) injected with DMSO for the first 7 days after cryoinjury and then with GM6001 for the following 7 days; or (c) injected with GM6001 for the first 7 days after cryoinjury and then with DMSO for the following 7 days (Fig. 2Ba,Bc). Our results show that the scar resolution at 30 dpc, presented as volume percentages of groups (a), (b), and (c) were ~1.7%, ~1.9%, and ~4.1%, respectively (Fig. 2Bd). Group (c), injected with GM6001 on the first week, had significantly larger scars than groups (a) and (b) with *p* values of 0.013 and 0.016, respectively. The scar volumes of groups (a) and (b) were comparable (*p* = 0.72; Fig. 2Bd). These results suggest that MMP activity might be most critical during the inflammatory phase (i.e., in the first 7 days) of heart regeneration.

### GM6001 affects the proliferation of cardiomyocytes and accumulation of fibroblasts

**Figure 3:**
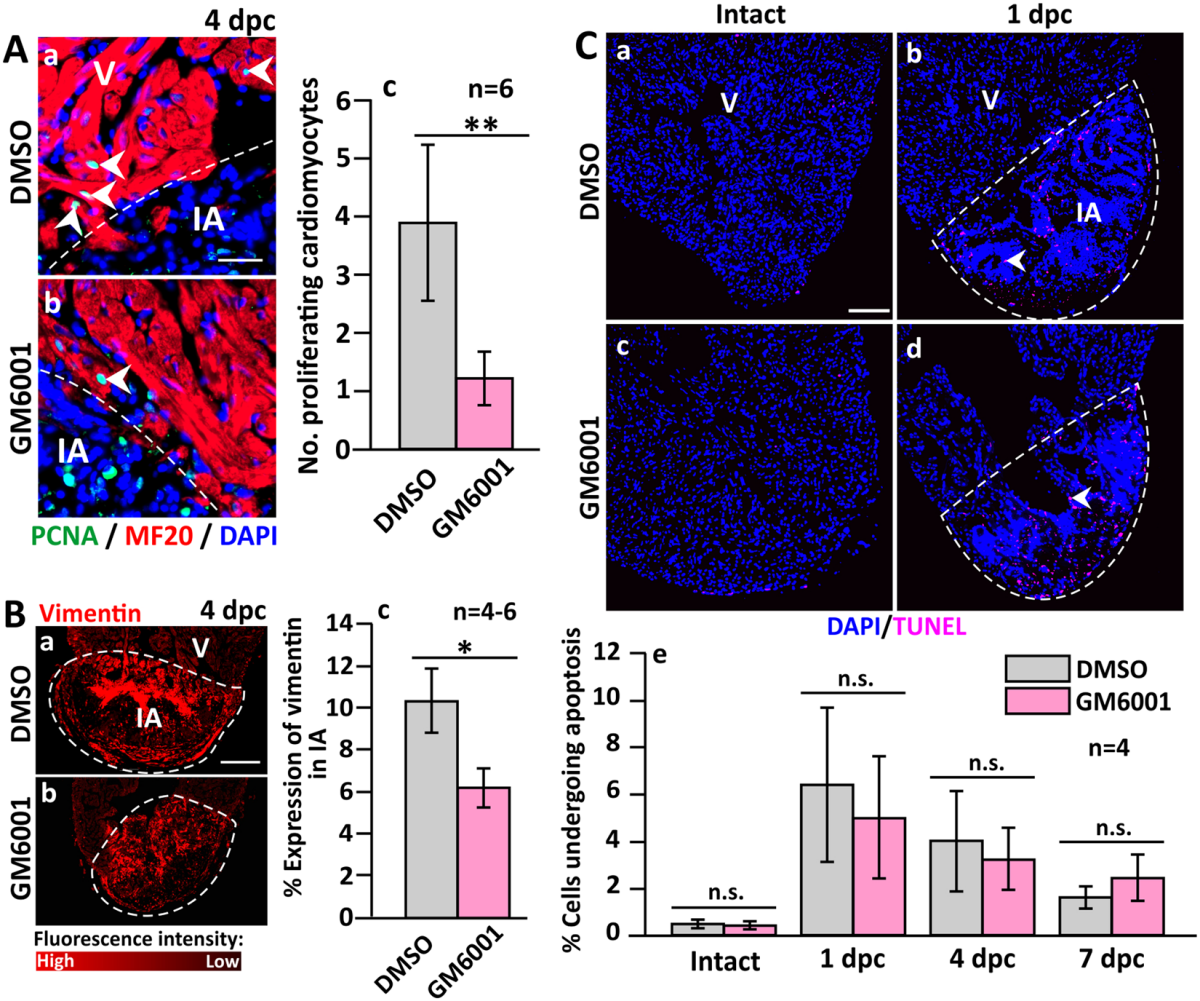
The number of proliferating cardiomyocytes and the accumulation of fibroblasts are reduced (but apoptosis is not affected) in GM6001-treated fish following cryoinjury. (**A**) Representative paraffin sections showing the margin between the intact ventricle and injured area (IA) in (Aa) DMSO control and (Ab) GM6001-treated fish, which were immunolabelled with MF20 and an anti-PCNA antibody to label the cardiomyocytes and proliferating cells, respectively. The sections were also co-labeled with DAPI to label the nuclei. (Ac) Bar chart to show the numbers of proliferating cardiomyocytes in the margin between the intact ventricle and IA in control and GM6001-treated fish. (Ba,Bb) Representative paraffin sections were immunolabelled with an anti-vimentin antibody to label fibroblasts, after which (Bc) the percentage of vimentin expression in the scar of the DMSO- and GM6001-treated fish at 4 dpc was determined. (**C**) Representative paraffin sections of intact and cryoinjured hearts after the fish were injected with either: (Ca,Cb) DMSO (controls) or (Cc,Cd) GM6001 (prepared in DMSO). Cells undergoing apoptosis were stained with TUNEL (pink). (Ce) Bar chart to show the percentage of apoptotic cells in the intact ventricle, and in the injured area at 1 dpc to 7 dpc. In (Ac and Bc), the data are expressed as the mean ± standard deviation of n = 4–6 hearts, and the asterisks indicate GM6001 data that are significantly different from the DMSO controls at p < 0.05 (*) and p < 0.01 (**), two-tailed t-test. In (Ce), the data are expressed as the mean ± standard deviation of n = 4 hearts, and n.s. indicates data that are not statistically different, one-way ANOVA with LSD Post-hoc test.

We also investigated if GM6001 treatment might lead to an inhibition in the number of proliferating cardiomyocytes. Thus paraffin sections were dual-immunolabeled with the myosin heavy chain antibody, MF-20 (to identify the cardiomyocytes), and an anti-PCNA antibody (to label the nuclei of proliferating cells), after which they were counterstained with DAPI to label the nuclei of all the cells (Fig. 3A). Since cardiomyocytes are reported to lose their function after cryoinjury, and do not express myosin heavy chain^5^, most of the proliferating cardiomyocytes were identified near the upper margin of the IA (Fig. 3Aa,Ab). Our data show that the number of proliferating cardiomyocytes per field of view in the control group was significantly greater than in the GM6001-treated group (Fig. 3Ac, *p* = 0.002), indicating that GM6001 treatment did lead to an inhibition in cardiomyocyte proliferation following cryoinjury.

Given that at 4 dpc, the amount of vimentin-positive labeling was at a peak (Fig. S1A), and the overlap observed between vimentin labeling and *mmp9*/*mmp13* expression suggests that the vimentin-positive cells might secrete MMPs (Fig. S1B), we investigated the effect of GM6001 on the accumulation of fibroblasts at this time point (Fig. 3B). In the DMSO control group, strong vimentin signals were observed in the epicardial layer and deep within the IA (Figs 3Ba and S1Ac). In the GM6001-treated group, however, the area of vimentin fluorescence was significantly less in both of these locations (*p* = 0.01, Fig. 3Bb,Bc). These results suggest that GM6001 might indeed delay the accumulation of vimentin-positive fibroblasts in the IA at 4 dpc.

As it has been reported that apoptosis is upregulated following cryoinjury^4^, we investigated the level of apoptosis in control and GM6001-treated groups at 1 dpc, 4 dpc and 7 dpc using the TUNEL assay (Fig. 3C). Our data appeared to show that the number of apoptotic cells at each time point was similar following GM6001 treatment as in the DMSO controls. This suggests that the impaired scar resolution observed following pharmacological inhibition of MMPs, was not due to increased apoptosis. However, due to the large variability in the data obtained, it is not possible to firmly conclude that the effect of GM6001 on apoptosis was negligible. Nonetheless, the similar percentage of apoptotic cells observed in the cryoinjured hearts following treatment with GM6001 or DMSO, suggests that the MMP inhibitor did not induce any obvious organ level toxicity. A similar finding has also been reported in a zebrafish model of retinotectal injury following treatment with GM6001^27^.

### Neutrophil and macrophage infiltration is inhibited by GM6001

**Figure 4:**
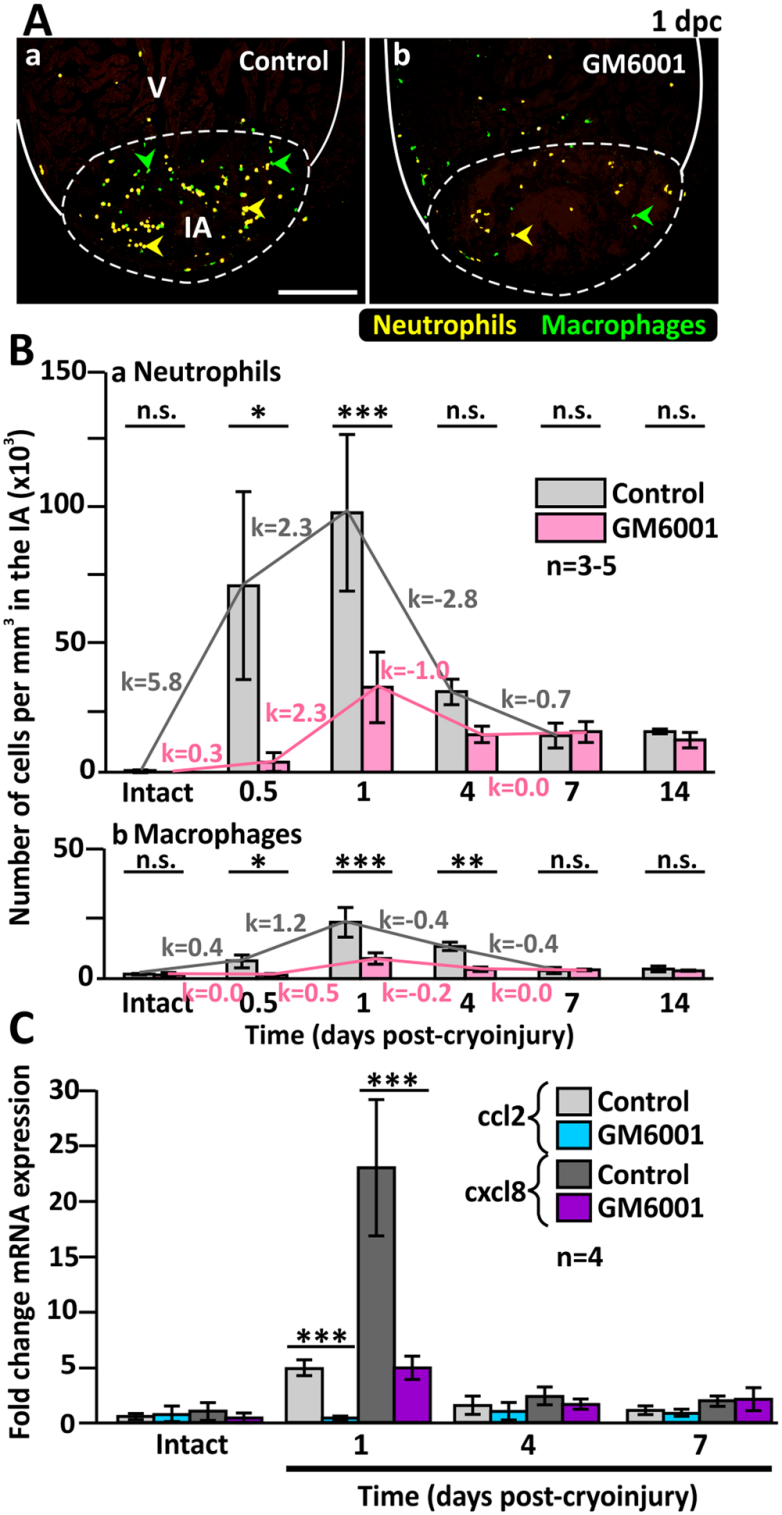
The numbers of inflammatory cells are reduced in the injured heart of GM6001-treated fish. (**A**) The Tg (*coro1a*:EGFP; *lyz*:dsRed) line of fish was used, where macrophages are labelled in green (see green arrowheads), and neutrophils are labelled in yellow (see yellow arrowheads). These images show the localization of macrophages and neutrophils in the injured areas (IA) of ventricles (V) at 1 dpc after the fish were injected with either (Aa) DMSO (controls) or (Ab) GM6001 (prepared in DMSO). The regions bounded by the white dashed lines indicate the IA, and scale bar: 200 µm. (**B**) Bar graphs to show the numbers of (Ba) neutrophils and (Bb) macrophages per mm^3^ in the IA of control and GM6001-treated fish at 0.5 dpc to 14 dpc, and in intact ventricles. The *k* values indicate the change in the numbers of cells per hour. (**C**) Bar graph to show the fold change in expression of *cxcl8* and *ccl2* in the intact heart and at 1 dpc, 4 dpc and 7 dpc in both control and GM6001-treated fish. In (**B**) and (**C**), the data are expressed as the mean ± standard deviation of n = 3–5 and n = 4 experiments, respectively, and the asterisks indicate GM6001 data that are significantly different from the DMSO controls at p < 0.05 (*), p < 0.01 (**) and p < 0.001 (***); one-way ANOVA with LSD Post-hoc test.

We used the double transgenic Tg (*coro1a*: EGFP; *lyz*: Dsred) zebrafish line^28^ to study the recruitment of neutrophils and macrophages into the IA of the heart after cryoinjury in GM6001-treated and DMSO control groups (Fig. 4). In this transgenic line, *coro1a*, which encodes the actin-binding protein, CORONIN 1a, and is specifically synthesized by leukocytes, is a marker for both neutrophils and macrophages, whereas *lyz* (encoding lysozyme) is a marker for neutrophils only. Thus, under a fluorescence microscope, neutrophils are yellow due to the co-localization of the red and green fluorescence signals, whereas macrophages are green (Fig. 4A). We profiled the densities of neutrophils and macrophages in the IA at 5 time points between 0.5 dpc to 14 dpc (Fig. 4B, Table S1). In intact hearts, there were no neutrophils and very few macrophages in the GM6001- or DMSO-treated groups. However, as early as 0.5 dpc, an infiltration of neutrophils and macrophages (i.e., ~69.7 × 10^3^ cells per mm^3^ and ~6.4 × 10^3^ cells per mm^3^, respectively) was found in the IA of the control group, whereas in the GM6001-treated group, significantly lower densities of both neutrophils and macrophages (i.e., with densities of ~3.7 × 10^3^ cells per mm^3^ and ~1.0 × 10^3^ cells per mm^3^, respectively) were found (*p* = 0.028, *p* = 0.00016). The density of neutrophils and macrophages in the IA was highest at 1 dpc for both groups. In the control group, the density of neutrophils and macrophages in the IA was ~97.7 × 10^3^ cells per mm^3^ and ~21.2 × 10^3^ cells per mm^3^, respectively, whereas in the GM6001-treated group, the density of neutrophils and macrophages was ~31.8 × 10^3^ cells per mm^3^ and ~7.4 × 10^3^ cells per mm^3^, respectively, which were significantly less than in the control group (*p* < 0.0001, *p* < 0.0001). At 4 dpc, the densities of macrophage remained significantly higher in the controls than in the GM6001-treated group (*p* = 0.001, *p* = 0.3, respectively). However, by 7 dpc and 14 dpc, no significant differences were observed between the control and GM6001-treated groups. Taken together, our data suggest that the pharmacological inhibition of MMP activity led to reduced leukocyte densities during the inflammatory phase, from 0.5 to 7 dpc. The speed of leukocyte recruitment was also impaired in the GM6001 group with the changes in leukocyte concentrations per hour (k values) being lower than those of the control group (Fig. 4B).

In zebrafish, CXCL8 and CCL2 are reported to guide the migration of neutrophils and macrophages, respectively^25,26^. Therefore, the expression of *cxcl8* and *ccl2* in the zebrafish heart were investigated by q-PCR (Fig. 4C). The level of expression of both *cxcl8* and *ccl2* was low in the intact heart, but both genes were upregulated significantly at 1 dpc, after which they returned to basal levels at 4 dpc and 7 dpc. GM6001 treatment resulted in the level of expression of both *cxcl8* and *ccl2* being significantly reduced, when compared with the control group at 1 dpc (*p* < 0.001, *p* < 0.001, respectively). In addition, our data showed that the peak of chemokine gene expression coincided with the peak of leukocyte density in the regenerating heart at 1 dpc (compare Fig. 4B with Fig. 4C), suggesting a potential role of MMPs in regulating chemokine expression, perhaps via a feedback mechanism.

Taken together, our data suggest that the recruitment of leukocytes, which is a critical step in the inflammatory phase of heart regeneration, was impaired when the enzymatic activities of MMP were inhibited pharmacologically.

### MMP9/MMP13 inhibitor I also reduced the heart regenerative capability

**Figure 5:**
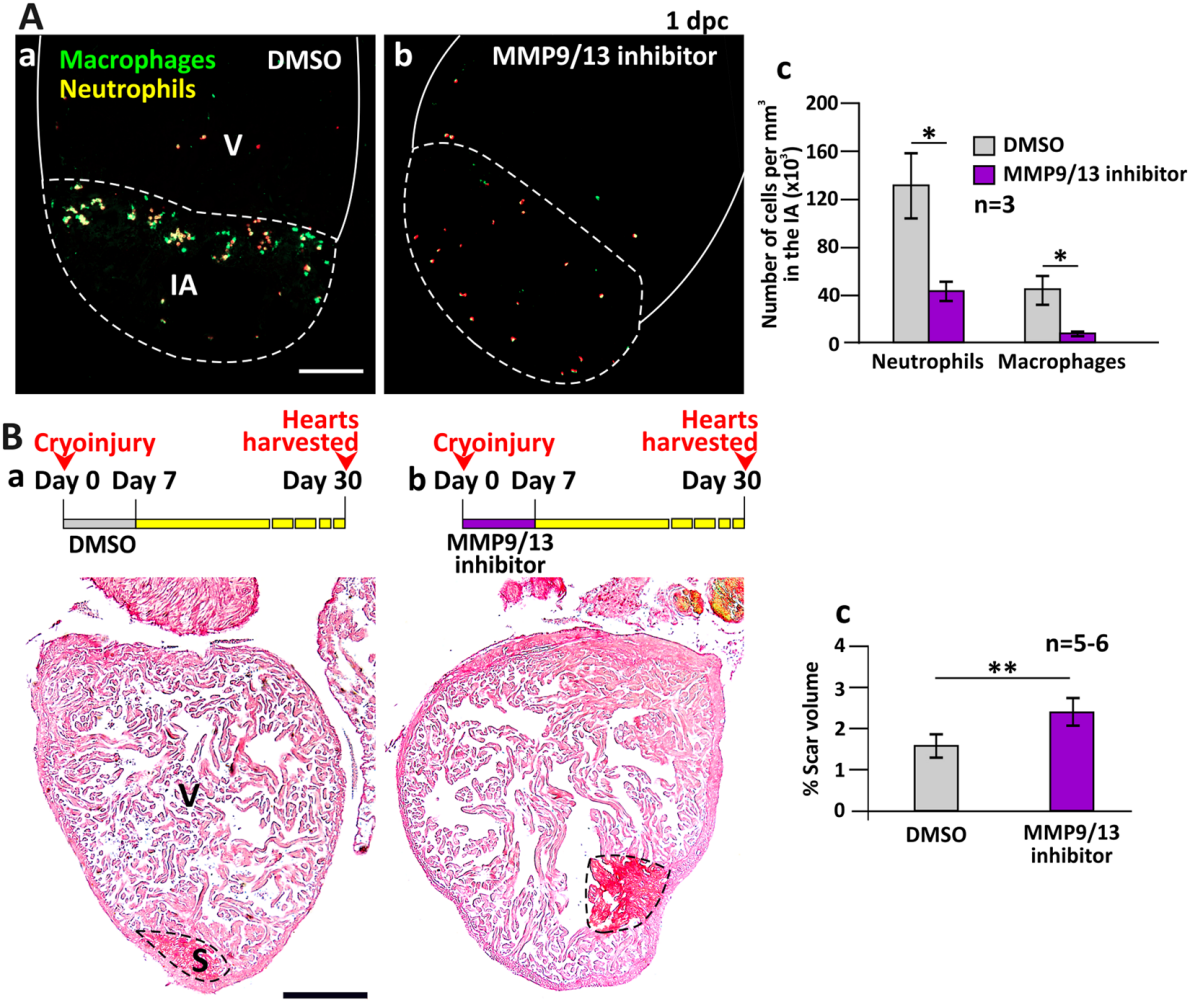
MMP9/13 inhibitor I (MMP9/13 inhibitor) reduced the number of inflammatory cells into the injured area (IA) and reduced the regenerative ability of the heart. (**A**) Tg (*coro1a*:EGFP; *lyz*:dsRed) fish were treated with DMSO (control) or MMP9/13 inhibitor after heart ventricle cryoinjury. These images show the localization of macrophages and neutrophils in the IA of ventricles (V) at 1 dpc. Scale bar: 200 µm. (Ac) Bar graph to show the numbers of neutrophils and macrophages per mm^3^ in the IA of control and MMP9/13 inhibitor-treated fish at 1 dpc. (**B**) Representative picrosirius red stained paraffin sections of wild type fish hearts harvested at 30 dpc, after the fish were injected with: (Ba) DMSO or (Bb) MMP9/13 inhibitor, every day for the first 7 dpc. Scale bar: 200 µm. (Bc) Bar graph to show the percentage of scar volume in the ventricle following DMSO or MMP9/13 inhibitor treatment. In (Ac) and (Bc), the data are expressed as mean ± standard deviation of n = 3 and n = 5 to 6 hearts, respectively. The asterisks indicate significantly different data at p < 0.05 (*) and p < 0.01 (**), two-tailed t-test.

It has previously been demonstrated via the use of a specific MMP9/MMP13 inhibitor^29^ that MMP9 (gelatinase B) and MMP13 (collagenase 3) together play a role in tumor cell invasiveness^30^, and that bone density defects can be rescued in a zebrafish model for osteoporosis^31^. To investigate their role in heart regeneration, we treated Tg (*coro1a*: EGFP; *lyz*: Dsred) zebrafish with MMP9/MMP13 inhibitor I (hereafter called MMP9/13 inhibitor; Fig. 5). At 1 dpc, when leukocyte recruitment was normally at a peak (Fig. 4), the densities of neutrophils and macrophages in the IA of the MMP9/13 inhibitor-treated group were ~43.6 × 10^3^ cells per mm^3^ and ~8.6 × 10^3^ cells per mm^3^, respectively (Fig. 5A). This is in comparison to the densities of these cells in the DMSO control group, which were ~131.7 × 10^3^ cells per mm^3^ and ~45.1 × 10^3^ cells per mm^3^, respectively. Thus, the specific inhibition of MMP9 and MMP13 significantly reduced the number of neutrophils and macrophages in the IA (*p* = 0.012, *p* = 0.013).

We also treated zebrafish with MMP9/13 inhibitor daily for the first 7 dpc and calculated the scar volume at 30 dpc (Fig. 5B). In the MMP9/13 inhibitor-injected group, the scar volume percentage was ~2.40%, which is significantly greater than that in the DMSO control group, which was ~1.58%, (*p* = 0.003). Thus MMP9/13 inhibitor reduced the number of leukocytes recruited and also reduced the heart regenerative capability following cryoinjury.

### Chemokine rescue of MMP-dependent leukocyte recruitment and regenerative capacity

**Figure 6:**
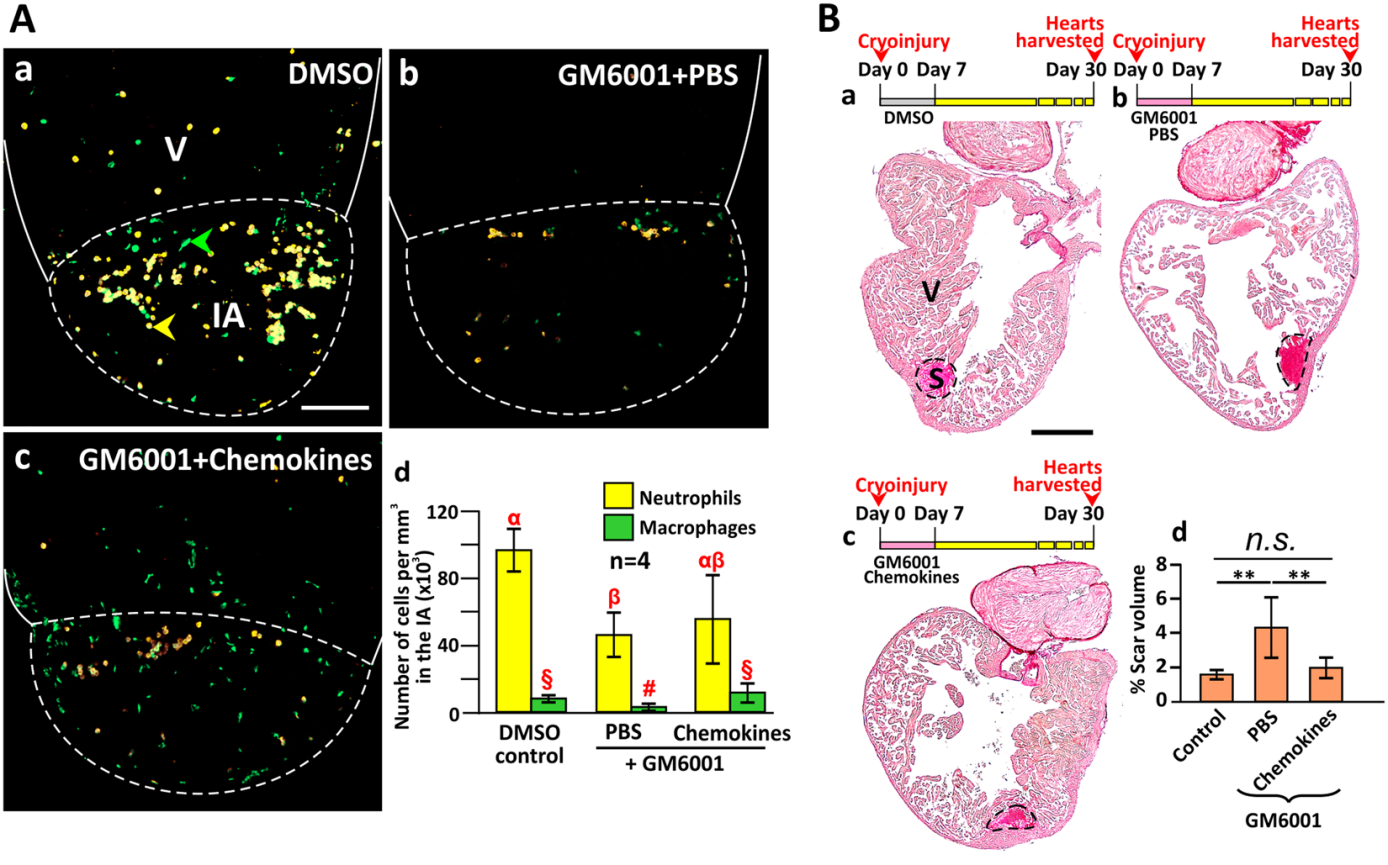
Injection of the chemokines CXCL8 and CCL2 rescued the impaired regeneration caused by GM6001. (**A**) The heart of Tg (*coro1a*:EGFP; *lyz*:dsRed) fish were cryoinjured and injected with (Aa) DMSO alone (control); (Ab) GM6001 and PBS; or (Ac) GM6001 and chemokines, after which the numbers of neutrophils (labeled in yellow) and macrophages (labeled in green) were quantified. Scale bar: 200 µm. (Ad) Bar graph to show the numbers (mean ± standard deviation) of neutrophils and macrophages per mm^3^ in the IA of fish at 1 dpc following the treatment regimens conducted in (Aa–Ac). Statistical analysis was carried out by One-way ANOVA with LSD Post-hoc test and significant differences (at *p* < 0.05) are shown by the different symbols (α, β, αβ and §, #) above the bars for the neutrophils and macrophages. (**B**) Wild type fish hearts were cryoinjured and then either (Bb) PBS or (Bc) chemokines were injected into the injured area following daily treatment with GM6001 during the first week after injury. (Ba) Some fish were injected with DMSO as a blank control. Serial paraffin heart sections were prepared and stained with picrosirius red. ‘V’ and ‘S’ are the ventricle and scar of the IA, respectively. Scale bar: 200 µm. (Bd) Bar graph to show the percentage of scar volume in the ventricle following the different treatments. The data are expressed as mean ± standard deviation of n = 5 to 6 hearts. The asterisks indicate significantly different data at *p* < 0.01, and ‘n.s.’ indicates results where no statistically significant differences were found, one-way ANOVA with LSD Post-hoc test.

Rescue experiments were conducted to investigate if CXCL8 and CCL2 might be substrates of MMP enzymes during the inflammatory phase of heart regeneration, as this would provide a link between MMP activity and leukocyte recruitment during heart regeneration (Fig. 6). To investigate if the defective leukocyte recruitment observed in the GM6001 treatment group might be effected via chemokines, zebrafish were: (a) treated with intraperitoneally with DMSO (as a control for GM6001); (b) injected intraperitoneally with GM6001 and into the IA with PBS (as the vehicle control) at 12 hpc and 18 hpc; or (c) injected intraperitoneally with GM6001 and into the IA with mixture of CXCL8 (250 μg/mL) and CCL2 (650 μg/mL) at 12 hpc and 18 hpc (Fig. 6A). We developed this treatment regimen because the highest rates (*k*-values) of leukocyte recruitment for both neutrophils and macrophages occurred from 0.5 dpc to 1 dpc (Fig. 4B). Hearts were collected at 24 hpc, and the neutrophils and macrophages in the IA were quantified (Fig. 6Ad). The densities of neutrophils for groups (a), (b) and (c) were 96.9 × 10^3^ cells per mm^3^, 47.3 × 10^3^ cells per mm^3^ and 56.2 × 10^3^ cells per mm^3^, respectively, and statistical analyses showed that chemokine rescue did not increase the recruitment of neutrophils significantly. In contrast, injection with CXCL8 and CCL2 significantly (*p* = 0.041) restored the number of macrophages in the IA, as the densities of macrophages in groups (a), (b) and (c) were 9.3 × 10^3^ cells per mm^3^, 4.1 × 10^3^ cells per mm^3^ and 12.9 × 10^3^ cells per mm^3^, respectively. These data indicate that injection of intact CCL2 was adequate in restoring the recruitment of macrophages. To investigate whether chemokine treatment alone might affect leukocyte recruitment following cryoinjury, zebrafish were: (a) used as a blank control without any injection; (b–e) injected into the IA at 12 hpc and 18 hpc with: (b) PBS (as the vehicle control); (c) CCL2 (650 μg/mL); (d) CXCL8 (250 μg/mL); or (e) a mixture of CXCL8 (250 μg/mL) and CCL2 (650 μg/mL). Hearts were then collected at 24 hpc, and the neutrophils and macrophages in the IA were quantified (Fig. S2). The densities of neutrophils and macrophages were similar in these 5 control groups, suggesting that the presence of additional chemokines did not impact upon leukocyte recruitment at 1 dpc in a regenerating zebrafish heart.

We also conducted experiments to study the effect of added CCL2 and CXCL8 to rescue the regenerative capability of the heart following GM6001 treatment (Fig. 6B). Zebrafish were injected: (a) with DMSO intraperitoneally for 7 days (as a control for GM6001; Fig. 6Ba); (b) with GM6001 intraperitoneally and with PBS into the IA (as a control for the chemokines) for 7 days (Fig. 6Bb); or (c) with GM6001 intraperitoneally and with chemokines into the IA for 7 days (Fig. 6Bc). After 30 days, the percentage of scar volume of groups (a–c) was ~1.58%, ~4.33%, and ~1.96%, respectively. Thus the scar volume of the GM6001/chemokine group was significantly lower than it was in the GM6001/PBS control group (*p* = 0.007), but it was similar to the DMSO control group (Fig. 6Bd). These data suggest that the concurrent injection of chemokines into the IA during the entire inflammatory phase was able to rescue the impaired regeneration caused by GM6001 treatment.

### MMP9 and MMP13 cleave CXCL8 at the same site

**Figure 7:**
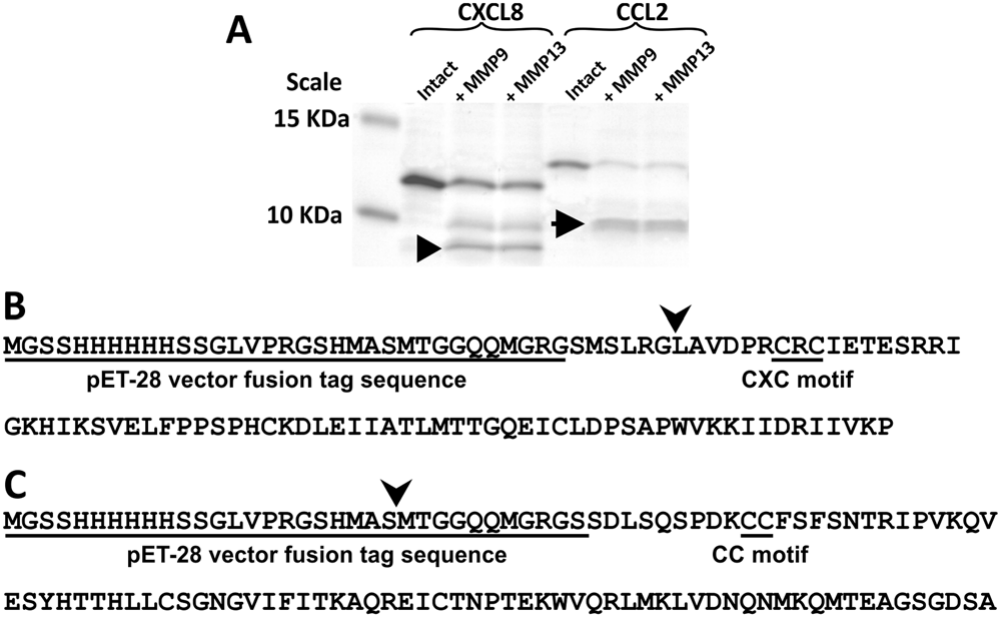
MMP-9 and MMP-13 cleave recombinant CXCL8 in the same way. (**A**) A representative Tricine-SDS-PAGE gel to show that recombinant CXCL8 and CCL2 were cleaved by MMP-9 and MMP-13. Two bands of similar sizes were generated after recombinant CXCL8 was cleaved by MMP-9 or MMP-13 (the smaller sized band is indicated by a black arrowhead). In contrast, just one band (arrow) was obtained after recombinant CCL2 was cleaved by MMP-9 or MMP-13. (**B**,**C**) N-terminal sequencing to show the cutting site of MMP-9- and MMP-13-cleaved (**B**) CXCL8 and (**C**) CCL2.

It has been reported that in humans, MMP9 cleaves CXCL8 and the resulting truncated form of the chemokine is 27-fold more chemotactic for neutrophils than the intact form^21^. In contrast, MMP9-cleaved CCL2 is 2-fold less chemotactic to monocytes^32^. To investigate how MMPs might process chemokines in zebrafish, recombinant CXCL8 and CCL2 were digested with MMP9 or MMP13, and a Tricine-SDS-PAGE was used to separate the products (Figs 7A and S3). The results show that MMP9 and MMP13 both cleaved recombinant CXCL8 in the same way, each resulting in the generation of two bands of the same sizes. In addition, MMP9 and MMP13 cleaved CCL2 in the same way as a single band (see black arrow) showing proteins of the same molecular weight was generated in each case. N-terminal sequencing of the cleaved forms of CXCL8 showed that both MMP9 and MMP13 cleaved CXCL8 at the fifth amino acid of the N-terminus (i.e., MSLRG↓LAVD; see arrowhead in Figs 7B and S4). In contrast, when the MMP9 or MMP13-treated product of recombinant CCL2 was sequenced, the first four amino acids were shown to be MTGG and thus part of the plasmid vector sequence (see arrowheads in Figs 7C and S5). Thus, unlike in humans, CCL2 in zebrafish was not cleaved directly by MMP9 or MMP13, perhaps due to a lack of cleavage sites.

### MMP9 activates CXCL8 *in vivo* and *in vitro*

**Figure 8:**
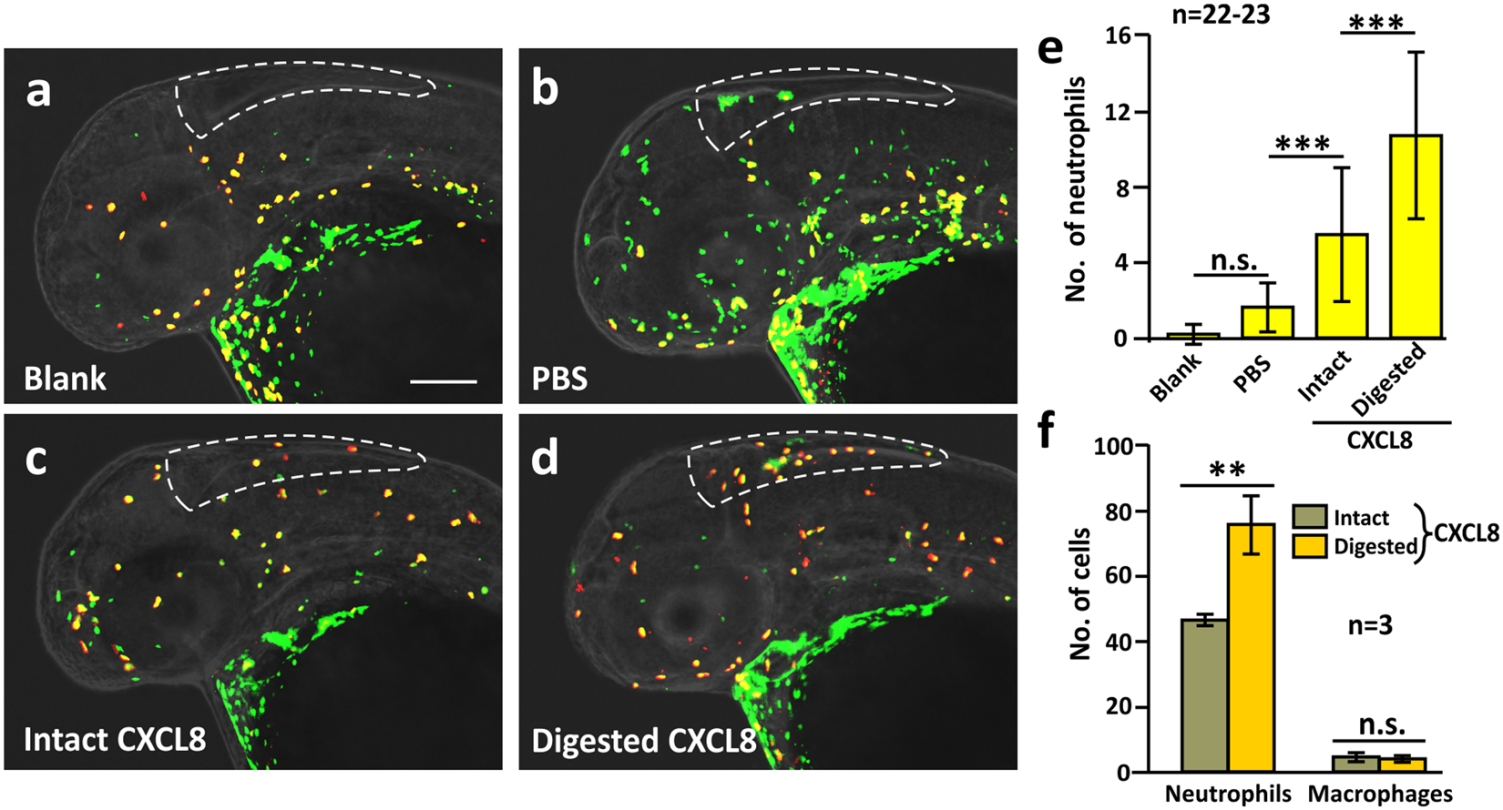
MMP-9-digested CXCL8 exhibited an increased ability to recruit neutrophils *in vivo*. Intact or MMP-9-digested CXCL8 was injected into the hindbrain of Tg (*coro1a*: EGFP; *lyz*: Dsred) zebrafish larvae at 2 dpf. After 90 min, the larvae were fixed and dual-immunolabeled with anti-GFP and anti-RFP antibodies, after which images were acquired by 3D high-resolution light-sheet microscopy under laser illumination. (**a**–**d**) Representative images to show the neutrophils (yellow) and macrophages (green) in the hindbrain of (**a**) blank or (**b**) PBS-injected control fish, as well as after injection of either (**c**) intact or (**d**) MMP-9-digested CXCL8. Scale bar: 200 μm. (**e**) Bar chart to show the mean ± standard deviation number of neutrophils in the hindbrain of zebrafish following the various treatments described above. The asterisks (***) represent significant differences when comparing the various groups at *p* < 0.001, and ‘n.s.’ indicates data that were not significantly different, one-way ANOVA with LSD Post-hoc test. (**f**) A cell suspension from 4 dpf larvae of the Tg (*coro1a*: EGFP; *lyz*: Dsred) line was prepared. An *in vitro* cell migration assay was performed and the numbers of neutrophils (yellow), and macrophages (green) were quantified. Bar chart to show the mean ± standard deviation numbers of neutrophils and macrophages that migrated towards intact or MMP-9-digested CXCL8. The asterisks (**) indicate intact and digested CXCL8 data that were significantly different at *p* < 0.01, and ‘n.s.’ indicates data that were not significantly different, two-tailed t-test.

To investigate if MMP9 cleavage might generate an active form of CXCL8, the intact and cleaved forms of the chemokine were injected into the hindbrain of Tg (*coro1a*: EGFP; *lyz*: DsRed) larvae at 2 days post fertilization (dpf) as an *in vivo* cell migration assay (Fig. 8). In blank control larvae (with no injection), very few neutrophils were found in the hindbrain at 2 dpf (Fig. 8a,e). After PBS (vehicle control) injection, the number of neutrophils increased (Fig. 8b,e). We suggest that this might be due to the physical trauma caused by the microinjection. In addition, larvae injected with CXCL8 digested by MMP9 for 24 h, exhibited a significantly higher number of neutrophils in the hindbrain than those injected with the intact form of the chemokine (Fig. 8c–e). This indicates the presence of a more chemotactic form of CXCL8 following MMP9 cleavage (Fig. 8d,e, *p* < 0.001). We also investigated the chemoattractive ability of intact and digested CXCL8 *in vitro* using a cell migration chamber and cells collected from Tg (*coro*1: EGFP; *lyz*: DsRed) larvae at 4 dpf (Fig. 8f). We showed that the digested form of CXCL8 was significantly more efficient (*p* = 0.009) at recruiting neutrophils than intact CXCL8, which was mixed with MMP9 just before the cell migration chamber was set up, with ~76 ± 9 cells and ~46 ± 2 cells migrating through the insert chambers, respectively. The migration of macrophages was used as an internal control, with the numbers of cells moving from the upper to the lower chamber remaining similar in both conditions (Fig. 8f). Taken together, our data show that the truncated form of CXCL8 in zebrafish was more chemoattractive than the intact form.

## Discussion

In zebrafish, heart regeneration involves a series of cellular and molecular events, which occur in a tightly controlled spatial and temporal manner. Based on various previous reports, it is now possible to describe the dynamic series of events that occur during heart regeneration following cryoinjury (where a scar is formed in a similar manner to that during mammalian myocardial infarction), as comprising 3 main phases. These are: 1) the inflammation phase, which occurs during the first 7 dpc; 2) the collagen deposition phase, which takes place from 7 dpc to14 dpc; and 3) the tissue remodeling and scar resolution phase, which occurs from 14 dpc to 60 dpc^5,17,20,22^. Here, we investigated if the enzymatic activity of MMPs might be essential for zebrafish heart regeneration following cryoinjury, especially with regards to the inflammatory phase during the first 7 days. We followed the recruitment of neutrophils and macrophages over time, and showed that the entry of both cell types happens during the first 24 hours post-cryoinjury. This resulted in peak densities of both cell types at 1 dpc, but homeostasis was restored by 7 dpc. Although it has previously been demonstrated that neutrophils and macrophages have distinct patterns of resident time^22^, our new data indicate that the recruitment and dispersal of both neutrophils and macrophages overlapped at the 6 time points tested. We also showed that the expression of both *cxcl8* and *ccl2* also peaked at 1 dpc, and so we tested whether the chemokines these genes encode might be target substrates of MMPs during this critical inflammatory period. We showed that concurrent injection of intact CXCL8 and CCL2 alleviated the impaired heart regeneration caused by MMP inhibition. We also showed that CXCL8 could be cleaved by MMP9 and MMP13, and the cleaved form was more chemoattractive to neutrophils than the intact form. In contrast, CCL2 was not cleaved by either of the MMPs tested.

Here, we focused on 3 important players of inflammation and resolution, namely MMPs, leukocytes and chemokines. In humans, ventricle damage due to myocardial infarction results in pathological scarring and insufficient regeneration of the heart^33^. In addition, most of the clinical trials conducted on novel therapeutic agents of cardiovascular diseases, which specifically target inflammatory modulation, have not elicited any drastic improvement in cardiac function nor relief of heart failure symptoms^34^. Hence, additional work is required to determine optimal doses and durations of current treatment regimens, as well as identification of novel therapeutic targets. Our data on the chemokine rescue of leukocyte recruitment to the IA is consistent with a previous report on the restoration of macrophage response in the diabetic wound following injection of intact CCL2^33^. Our work with zebrafish demonstrates that the MMP-mediated leukocyte recruitment, which occurs via the cleavage of chemokines, is a tightly orchestrated series of events, which is essential for a successful outcome of heart regeneration. In future experimentation on mammalian models of myocardial infarction, it would be interesting to test specific combinations of MMPs (such as MMP9 and MMP13), as well as restricting the period of intervention to that of the inflammatory period only. With MMPs exerting both positive and negative actions on the infarct, it might be that the timing of intervention is crucial for promoting a favorable outcome.

We also showed that cleaved CXCL8 enhanced the recruitment of neutrophils. This together with the peak but transient expression of *mmp9* and *mmp13* in vimentin-positive fibroblasts at 4 dpc (Figs 1 and S1), suggests that a positive feedback mechanism of MMP transcription, translation and enzymatic activities exist in zebrafish, similar to that reported in humans^21^. With regards to CCL2, in humans this chemokine can be cleaved by MMP9; however in its cleaved form, CCL2 is less chemotactic^32^. We suggest that without the cleavage and hence the inactivation of CCL2, macrophage recruitment in zebrafish continues at the same level throughout the entire inflammatory and resolution phases. Indeed, it has previously been shown that delayed macrophage recruitment in zebrafish results in impaired heart regeneration as measured by the rate of neovascularization, the speed of neutrophil clearance, and the rate of cardiomyocyte proliferation and scar resolution^21^. In the future, therapeutic agents that promote the recruitment of macrophages (changing the timing of their entry and residence time) might be explored.

While the current experiments were focused around the use of zebrafish as an animal model, our results might also provide new information about the function of MMPs during the inflammatory phase following heart injury in other vertebrates. In addition, they might provide new insights for the future development of chemokine-related therapies in mammals, to aid in the repair of diseased and damaged tissues and organs. Indeed, a range of chemokine-based immunotherapies are already starting to be developed for the treatment of cancer and these might well be re-purposed for cardiovascular diseases^35^.

## Methods

### Zebrafish maintenance

In the following experiments, the Tg (*coro1a*: EGFP; *lyz*: Dsred) line of transgenic zebrafish (*Danio rerio*), and the wild type AB strain of fish were used. The AB strain was acquired from the Zebrafish International Resource Center (ZIRC; University of Oregon, Eugene, OR, USA); whereas the Tg (*coro1a*: EGFP; *lyz*: Dsred) line was provided by Prof. Zi-Long Wen (HKUST, Hong Kong). The fish were maintained in a controlled environment as described by Liu *et al*.^36^. All the animal procedures used in this study were approved by the Department of Health, Hong Kong, SAR, China (refs (14–118) in DH/HA&P/8/2/5 Pt.3), and the experiments were conducted in accordance with the relevant guidelines and regulations in Hong Kong, SAR, China.

### Cryoinjury of the zebrafish heart

Zebrafish (6–12 months old) were individually anesthetized in 0.05% MS-222 (E10521; Sigma-Aldrich). The fish heart was exposed, and cryoinjury was conducted on the ventricle, using methods described by Chablais *et al*.^8^. The sham controls comprised fish in which the heart was not subjected to cryoinjury following an incision of the chest.

### *In situ* zymography

Zebrafish hearts were harvested and embedded with OCT gel (Leica Biosystems). The embedding blocks were quick-frozen in liquid nitrogen, and 10-μm cryosections were prepared. Gelatinolytic activity was demonstrated by incubating the sections with DQ-gelatin (EnzChek; Molecular Probes) at 28 °C for 10 min in the dark. The DQ-gelatin was removed and the sections were fixed (and MMPs inactivated) with 4% paraformaldehyde (PFA) for 10 min. The sections were then washed with PBS, and mounted under coverslips with PBS. Images were taken using an Olympus BX61 microscope and DP72 digital camera.

### RNA probe synthesis and *in situ* hybridization (ISH)

The PCR products of *mmp9* (accession numbers: AY151254) and *mmp13* (accession numbers: AF506756) were used as templates to synthesize RNA probes. The primers for *mmp9* and *mmp13* were as follows: Ish-mmp9-F: 5′-ACCACCGCAGACTATGACAA-3′, Ish-mmp9-R: 5′-TAATACGACTCACTATAGGGACCTTTGCGTTCACCATT-3′; Ish- mmp13-F: 5′-GACT CGACTGTATGGGTTGC-3′, Ish-mmp13-R: 5′-TAATACGACTC ACTATAGGGTTGAATCATCGGTATTGACG-3′. Digoxigenin-labeled antisense RNA probes were generated using the Roche RNA labeling kit (Roche Diagnostics Ltd.) according to the manufacturer’s instructions, and *in situ* hybridization was performed according to Lien *et al*.^17^.

### Histological techniques

Zebrafish were sacrificed and the heart was surgically excised and fixed with 4% PFA at 4 °C overnight. Paraffin sections of ~5 μm thickness were prepared. Picrosirus red staining (ab150681, Abcam) was performed to determine the volume of the scar in the zebrafish heart in GM6001-treated and control groups. For morphometric analysis, bright-field images were acquired of every fourth section using the microscope and digital camera described above. For each heart sample, the percentage of the scar volume with respect to the volume of the whole ventricle was calculated according to the equation below:

$${\rm{Percentage}}\,{\rm{of}}\,{\rm{scar}}\,{\rm{volume}}=\frac{\sum _{n}^{i}Si}{\sum _{n}^{i}Vi}\times 100 \% $$

Where *n* is the total number of sections; *i* is the section number; *Si* is the area of the scar of the i^th^ section; and *Vi* is the area of the ventricle of the i^th^ section.

Image processing was conducted with Image J (National Institutes of Health, Bethesda, MA, USA).

### GM6001 and MMP9/MMP13 inhibitor I treatment, and chemokine injection

GM6001 (ab120845; Abcam) and MMP9/MMP13 inhibitor I (21265; Cayman) were prepared at a stock concentration of 20 mM in DMSO. Just prior to use, the GM6001 or MMP9/MMP13 inhibitor I stock solution was diluted to 100 μM with Hanks’ solution. Zebrafish were anesthetized and then injected intraperitoneally with 10 μL of the 100 μM GM6001 or MMP9/MMP13 inhibitor I solution. A control group was injected with 10 μL Hanks’ solution containing 0.5% DMSO alone. In one series of experiments, zebrafish were injected with GM6001 (or DMSO) once a day for up to 30 days. In another series of experiments, zebrafish were split into three groups: one group was injected with DMSO alone for 14 days; another group was injected with GM6001 for 7 days and DMSO for the following 7 days; and the final group was injected with DMSO for 7 days and then with GM6001 for the next 7 days. Immediately after the first injection, the heart was cryoinjured, as described above.

In one series of experiments, following cryoinjury and GM6001 treatment, the densities of leukocytes and the expression of the *cxcl8* and *ccl2* genes were measured at various time points. Intact hearts were harvested from fish treated for 24 hours with DMSO or GM6001 and used as the control group.

In another series of experiments, following cryoinjury and GM6001 treatment, anesthetized zebrafish were microinjected into the injured area of the ventricle with a ~15 nL mixture of CXCL8 and CCL2 (at 250 μg/mL and 650 μg/mL in PBS, respectively). Zebrafish were injected with these chemokines twice a day from 1 dpc to 4 dpc, and then once a day from 5 dpc to 7dpc. Control fish were cryoinjured and treated with GM6001, but injected with PBS only. In an additional series of control experiments, cryoinjured fish without GM6001 treatment were injected with CXCL8 and CCL2 individually or in combination into the injured area. The hearts were harvested at different time points, and the volume of the scar as well as the numbers of neutrophils and macrophages were quantified.

### Immunohistochemistry

Paraffin sections of cryoinjured hearts were deparaffinized and antigen retrieval was performed by incubating the sections in sodium citrate buffer (10 mM sodium citrate, 0.05% Tween 20, pH 6.0) at 95 °C for 15 min. The sections were then rinsed with distilled water, and washed with PBST (PBS containing 0.125% Triton X-100), after which they were incubated in blocking buffer (PBST containing 1% BSA and 10% goat serum) for 1 h at room temperature. The following primary antibodies were used: mouse anti-vimentin (ab8978; Abcam); rabbit anti-GFP (ab13970; Abcam); mouse anti-RFP (ab62341; Abcam); rabbit anti-PCNA (sc-7907; Santa Cruz); or mouse anti-myosin heavy chain (MF-20; DSHB). The secondary antibodies used were Cy3-conjugated goat anti-mouse (A10521; Invitrogen) or Alexa Fluor 488-conjugated goat anti-rabbit (A11034; Invitrogen) antibodies. Fluorescence images were acquired using the microscope and camera described above. The number of proliferating cardiomyocytes was quantified per field of view under 20× magnification at the edge of the injured area. The area of vimentin expression was quantified in the injured area using Image J and the percentage of vimentin expression with respect to the size of the injured area was calculated. The area of the scar was determined, and the numbers of neutrophils and macrophages in four representative sections in the injured area were quantified. The density of neutrophils and macrophages was determined by calculating the (total number of neutrophils or macrophages)/(injured area × thickness).

### Terminal deoxynucleotidyl transferase dig-dUTP nick end-labeling (TUNEL)

Paraffin sections were prepared and deparaffinized. The cells undergoing apoptosis were detected using the DeadEnd™ Fluorometric TUNEL System kit (G3250; Promega) according to the manufacturer’s instructions. Images were acquired using the microscope and camera described above, and the percentage of apoptotic cells in the ventricle and injured area were calculated with Image J.

### Quantitative PCR (q-PCR)

Total RNA was extracted using Trizol reagent (9109; Takara Bio. Inc.), and cDNA was synthesized using the PrimeScript reverse transcription (RT) reagent kit (6210B; Takara Bio. Inc.) according to the manufacturer’s instructions. The expression of *cxcl8* (rtcxcl8F: 5′-GTCGCTGCATTGAAACAGAA-3′, rtcxcl8R: 5′-CTTAACCCATGGAGC AGAGG-3′), and *ccl2* (rtccl2F:5′-GTCTGGTGCTCTTC GCTTTC-3′, rtccl2R: 5′-TGC AGAGAAGATGCGTCGTA-3′) was determined by q-PCR using the SYBR Premix Ex Taq (RR402A; Takara Bio. Inc.), and *β-actin* (β-actin-F: 5′-GCTGACAGGATGCAGAA GGA-3′, β-actin-R: 5′-TAGAAGCATTTGCG GTGGAC-3′) was used as the reference gene. q-PCR was performed in triplicate for each gene, and the results were analyzed using the ΔΔ^CT^ method.

### Enzymatic cleavage of MMP9 and MMP13

Recombinant MMP9, MMP13, CXCL8 and CCL2 were produced in *E.coli*. (see supplementary methods). Mature CXCL8 and CCL2 (i.e., without the signal peptide at the N-terminus) were also synthesized by Zhejiang Ontores Biotechnologies Inc. (Zhejiang, China). Recombinant MMP9 and MMP13 were used to digest recombinant CXCL8 and CCL2 (at 400 μg/mL and 200 μg/mL, respectively) overnight at 37 °C. The digestion products were then separated via 16.6% Tricine–SDS-PAGE^37^, and the separated protein bands were stained with 0.1% Coomassie blue R-250.

### N-terminal sequencing of the cleaved chemokines

Following gel electrophoresis of the CXCL8 and CCL2 digestion products, the separated proteins were transferred from the gel to a PDVF membrane using 10 mM 3-[cyclohexylamino]-1-propanesulfonic acid buffer (Sigma-Aldrich; in 20% methanol, pH 11.0). The membrane was then stained with 0.1% Coomassie blue for 40 sec, followed by washing with 10% acetic acid and 30% ethanol to reduce the level of background staining. The region of the PDVF membrane containing the target band was excised and washed thoroughly with distilled water, after which it was dried between two pieces of filter paper and stored at 4 °C. The samples were then sent to Sangon Biotech (Shanghai) Co. Ltd. (Shanghai, China), for N-terminal sequencing of the truncated peptides by the Edman degradation method.

### *In vivo* neutrophil recruitment by synthesized chemokines CXCL8

The Tg (*coro1a*: EGFP; *lyz*: Dsred) line of fish was used in these experiments. When embryos reached 1 day post-fertilization (dpf), ~200 µM phenylthiourea (PTU; Sigma-Aldrich) was added to the culture water to inhibit the formation of pigmentation. At 2 dpf the fish larvae were injected with 1 nL of digested (200 μg/mL) or undigested CXCL8 (200 μg/mL), or with PBS into the hindbrain. Synthesized CXCL8 was digested with recombinant MMP9 as described above, whereas the undigested group was prepared by mixing CXCL8 with recombinant MMP9 just prior to injection. After 1.5 h, the larvae were fixed with 4% PFA for 2 h at room temperature. They were then washed with PBS, after which they were immunolabeled with anti-GFP and anti-RFP antibodies. The larvae were mounted in a glass capillary tube with 1% Low Melt agarose (50080; Lonza, Basel, Switzerland) and images of the head of the larvae were acquired using a Zeiss Lightsheet Z.1 3D microscope with a 5× water lens and the z-stack function. At the end of image acquisition, the left and right images collected were merged and deconvolution of each merged layer was performed, after which the images were exported using the maximum projection method and the numbers of neutrophils and macrophages in the hindbrain were quantified.

## Electronic supplementary material

Supplementary infromation
