# Supplementary material for: Matrix metalloproteinases (MMPs) mediate leukocyte recruitment during the inflammatory phase of zebrafish heart regeneration
Source: Sci Rep. 2018 May 8;8:7199. doi: 10.1038/s41598-018-25490-w (PMC5940908; doi:10.1038/s41598-018-25490-w)
Supplement: Supplementary file 1 — Supplementary infromation [file 41598_2018_25490_MOESM1_ESM.pdf]

Matrix metalloproteinases (MMPs) mediate leukocyte recruitment during the inflammatory phase of zebrafish heart regeneration.

Shisan XU[1], Sarah E. WEBB[1,2], Terrence Chi Kong LAU[1] and Shuk Han CHENG[1,3,4],*

[1] Department of Biomedical Sciences, City University of Hong Kong, Kowloon Tong, Kowloon, Hong Kong SAR, PRC.

[2] Division of Life Science, The Hong Kong University of Science and Technology, Clear Water Bay, Kowloon, Hong Kong SAR, PRC.

[3] State Key Laboratory of Marine Pollution (SKLMP) at City University of Hong Kong.

[4] Department of Materials Science and Engineering, College of Science and Engineering, City University of Hong Kong, Kowloon Tong, Kowloon, Hong Kong SAR, PRC.

*Author for Correspondence:

Prof. Shuk Han Cheng

Tel: (852) 3442-9027 / Fax: (852) 3442-0549 / E-mail: bhcheng@cityu.edu.hk

Supplementary Materials

Experimental Procedures

Immunolabeling cryosections with an anti-vimentin antibody

10 µm cryosections of zebrafish hearts were prepared as described in the Materials and Methods section on "In situ *zymography*". The sections were fixed with 4% PFA at room temperature for 30 min, and then washed with PBST (PBS containing 0.125% Triton X-100), after which they were incubated in blocking buffer (PBST containing 1% BSA and 10% goat serum) for 1 h at room temperature. The sections were incubated in primary antibody (mouse anti-vimentin, ab8978; Abcam) at 4°C overnight, and then washed with PBST, after which the sections were incubated in secondary antibody (Cy3-conjugated goat anti-mouse, A10521; Invitrogen) at room temperature for 2 h. Fluorescence images were acquired using the microscope and camera described in the Materials and Methods section on "*Immunohistochemistry*".

Dual in situ *hybridization and immunolabeling*

Fluorescence *in situ* hybridization and immunofluorescence co-labeling were performed on the same paraffin sections at 4 dpc. Thus, *mmp9* and *mmp13 in situ* hybridization was conducted as described in the Materials and Methods section on "RNA probe synthesis and *in situ* hybridization (ISH)", but the paraffin sections were blocked for 2 h in blocking buffer, after which peroxidase-conjugated anti-DIG (Fab fragments; at a dilution of 1:100 in blocking buffer) was used as the primary antibody, and the sections were incubated for 1 h at room temperature. An anti-digoxigenin-POD (Fab fragments; 11207733910; Roche) was used as the secondary antibody, after which the signals were visualized with the TSA plus Cy3 system (PerkinElmer Inc.), according to the manufacturer's instructions. Antigen retrieval and immunostaining were then performed as described in the Materials and Methods section on "Immunofluorescence labeling" with the mouse anti-vimentin primary antibody, and an Alexa Fluor 633-conjugated goat

anti-mouse (A21052; Invitrogen) secondary antibody.

Recombinant protein expression and purification

The protein coding sequences of MMP9, MMP13, CXCL8, and CCL2 were cloned by PCR. The following primers were used:

Ex-mmp9-F: 5'-CGGGATCCCCACTTAAATCTGTGTTCGTG-3'

Ex-mmp9-R: 5'-CCCAAGCTTTTAGGATGTCGAAGGTCTAT-3'

Ex-mmp13-F: 5'-CGGA ATTCCTGACTCGACTGTATGGGTT-3'

Ex-mmp13-R: 5'-ACGCGTCGACGATTCTTCTT CAGGCGGTAA-3'

Ex-cxcl8-F: 5'-CGGGATCCATGAGCTTGAGAGGTCTGG-3'

Ex-cxcl8-R: 5'-GTACCCAAGCTTCTTGACTTCACAGGTGATCC-3'

Ex-ccl2-F: 5'-CGGGAT CCAGTGATTTGTCCCAGAGTCC-3'

Ex-ccl2-R: 5'-TGACGCGTCGACGGACTGTTCC CATCTTAGGC-3'.

The PCR products of the MMP9, CXCL8 and CCL2 coding sequences and the corresponding pET-28α vectors were then double-digested with BamHI (R0136S) and HindIII (R0104S), whereas the MMP13 coding sequence and its corresponding pET-28α vector were double-digested with EcoRI (R0101S) and SalI (R0138S). All the restriction enzymes were from NEB Inc.. The digested coding sequences of each of the genes and the vectors were ligated with T4 ligase (M0202S; NEB Inc.), according to the manufacturer's instructions. The positive plasmids of the pET-28α-MMP9, pET-28α-MMP13, pET-28α-CXCL8 and pET-28α-CCL2 were identified by DNA sequencing at Tech Dragon Ltd. (Shatin, Hong Kong SAR).

Recombinant MMP9, MMP13, CXCL8 and CCL2 were induced with 1 mM IPTG at 37 ℃ in *E. coli BL21*, and purified using nickel magnetic beads (LSKMAGH02; Millipore), according to the manufacturer's instructions. Endotoxin removal resin (88270; Thermo Scientific) was then used to remove potential lipopolysaccharide (LPS) contamination, again according to the manufacturer's instructions. An Amicon Ultra-4

centrifugal filter device (UFC801008 and UFC800308; Millipore) was used to concentrate the recombinant proteins and remove any remaining imidazole. The recombinant proteins were finally dissolved in PBS. The enzymatic activity of MMP9 and MMP13 was measured using EnzChek DQ-gelatin (E12055; Molecular Probes), according to the manufacturer's instructions.

In vitro *cell migration*

A whole larval cell suspension was prepared from the Tg (*coro1a*: EGFP; *lyz*: Dsred) line of fish as previously described [1-2]. In these transgenic fish, neutrophils are labeled with both EGFP and dsRed, whereas macrophages are labeled with EGFP alone; thus it is possible to identify both of these cell types in the fish and also differentiate between the two. Approximately 200 larvae at 7 days post-fertilization (dpf), were rinsed with distilled water containing 5% bleach. The larvae were then washed with sterilized PBS, after which they were digested with 2 mL lysis buffer, (1× PBS, 10 mM HEPES, 30 mM taurine, 5.5 mM glucose, 5 mg/mL collagenase type II and 5 mg/mL collagenase type IV) at 32 ℃ with gentle, constant shaking until no large pellets remained. The digested mixture was then filtered through a 70 μm cell strainer (Falcon) to remove any large debris, and the reaction was stopped by adding five-fold the volume of L-15 medium (Gibco), containing 5% FBS and 200 U/mL penicillin-streptomycin. The cells were collected by centrifugation at 300 g for 5 min at 4 ℃. The supernatant was discarded and the cell pellet was resuspended in 1 mL fresh L-15 medium. The final cell concentration was adjusted to ~2×10^6 cells/mL.

In vitro cell migration assays were performed using a 6.5 mm Transwell with a 5 μm-pore size polycarbonate membrane insert (Corning Incorporated Life Sciences). Synthesized CXCL8 (400 μg) was digested by incubation with 5 μL recombinant MMP9 (~20 U/mL) in a total reaction volume of 50 μL at 37 ℃ overnight. Approximately 600 μL L-15 medium (containing 200 μg/mL of the MMP9-digested chemokine) was then

added to a 24-well plate. As a control, CXCL8 was mixed with MMP9 just before the migration assay was set up; thus the time and temperature required for cleavage were not provided. A Transwell insert was placed into each well and the 24-well plates were incubated at 28 ℃ for 2 h, after which 100 μL cell suspension was applied to the upper chamber of each well. Cells were incubated in this cell migration plate set-up at 28 ℃ for 4 h, after which photomicrographs of the cells that had migrated to the lower chamber were acquired using a Nikon Eclipse Ti-E Live Cell Imaging System. Images were acquired sequentially with the Cy3 (red) and FITC (green) filters, after which the red and green images were merged, and the numbers of neutrophils (which express dsRed and EGFP, and thus show up as yellow), and macrophages (which just express EGFP, and thus show up as green), were quantified.

References

[1]. Sander, V., Suñe, G., Jopling, C., Morera, C. & Belmonte, J. C. I. Isolation and in vitro culture of primary cardiomyocytes from adult zebrafish hearts. *Nat. Protoc*. **8**, 800-809 (2013).

[2]. Mustafa, A. & Dhawale, S. Development of a method for extracting macrophages from zebrafish, Danio rerio and their use to assess stress. *Acta Ichthyol. Piscat*. **38**, 1. doi: 10.3750/AIP2008.38.1.11 (2008).

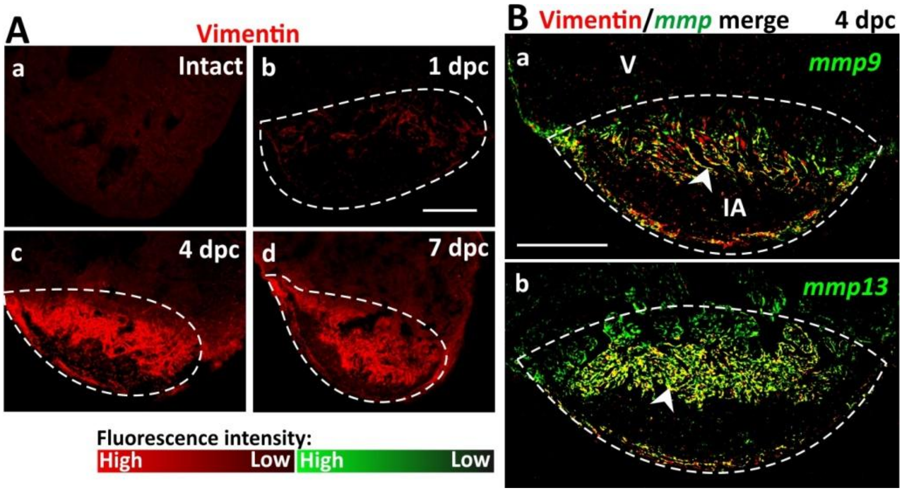

Figure S1. Elevated numbers of vimentin-positive fibroblasts in the injured area contribute to MMP production. (A) Immunofluorescence images of cryosections to show the localization of vimentin (in red) and hence the distribution of fibroblasts in (Aa) intact (control) and (Ab-Ad) cryoinjured wild type heart ventricles (V) at: (Ab) 1 dpc, (Ac) 4 dpc, and (Ad) 7 dpc. (B) Fluorescence *in situ* hybridization (FISH) was conducted to show the expression of (Ba) *mmp9* and (Bb) *mmp13* (in green), after which immunohistochemistry was performed to show the localization of vimentin (in red) in the IA of wild type heart ventricles at 4 dpc. These representative images of paraffin sections show the FISH and immunofluorescence data when merged. Regions of co-localization appear in yellow (see white arrowheads). In each panel, the regions bounded by the dashed white lines indicate the IA. Scale bars: 200 μm.

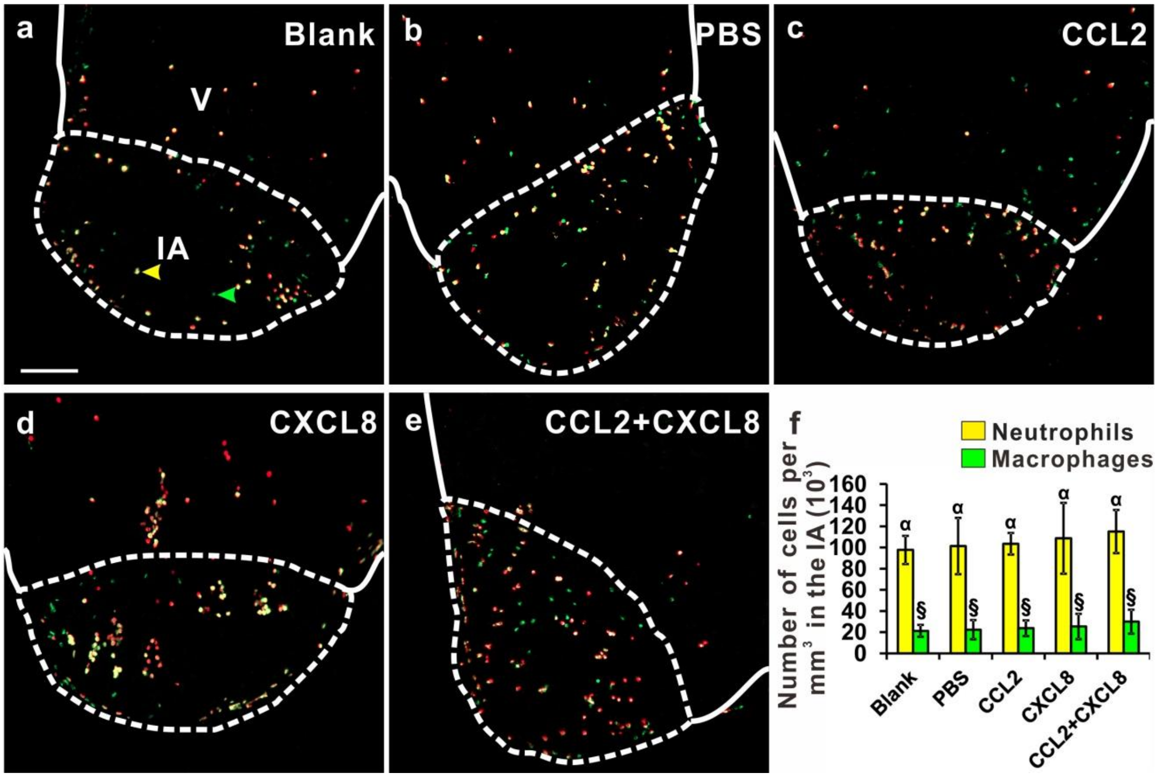

Figure S2 Injection of the chemokines did not affect the number of inflammatory cells in the injured area (IA). Using the Tg (*coro1a*:EGFP; *lyz*:dsRed) line of fish, the heart was cryoinjured and either: (a) not injected (as a blank control), or else injected with: (b) PBS; (c) CCL2; (d) CXCL8; or (e) CCL2 and CXCL8, after which the numbers of neutrophils (labeled in yellow) and macrophages (labeled in green) were quantified. Scale bar, 200 μm. (f) Bar graph to show the numbers (mean ± standard deviation, n= 5 to 6 hearts) of neutrophils and macrophages per mm^3 in the IA of fish at 1 dpc following the treatment regimens conducted in (a-e). The number of neutrophils in cryoinjured hearts following the treatments described in (a-e) were 95.7 ± 13.3, 101.4 ± 9.1, 103.6 ± 10.1, 108.7 ± 33.5 and 115.1 ± 20.4 $\times 10^3$ cells per mm^3, respectively; and the number of macrophages were 20.2 ± 5.6, 22.3 ± 9.4, 23.7 ± 7.4, 25.4 ± 12.0 and 29.9 ± 11.3 $\times 10^3$ cells per mm^3, respectively. Statistical analysis was carried out by One-way ANOVA and significant differences (at $p<0.05$) are shown by the different symbols above the bars.

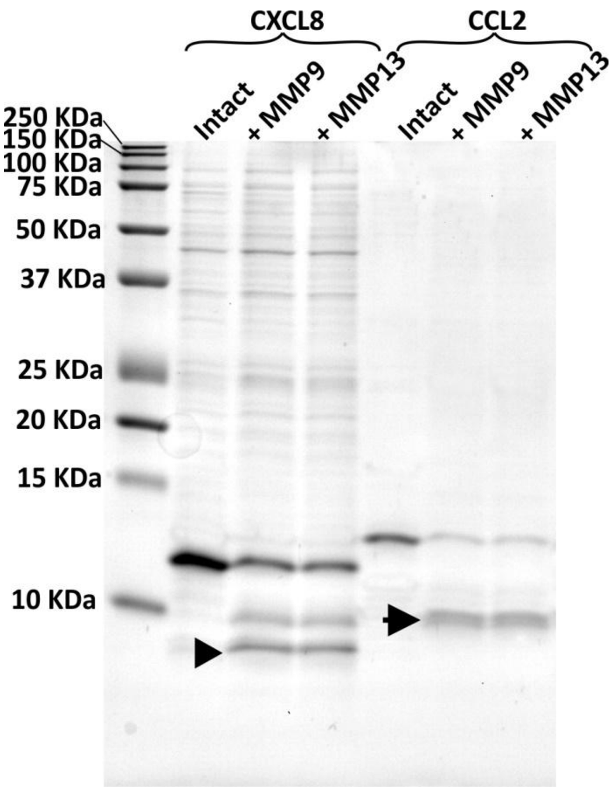

Figure S3 A representative Tricine-SDS-PAGE full-length gel to show that recombinant CXCL8 and CCL2 were cleaved by MMP-9 and MMP-13. Two bands of similar sizes were generated after recombinant CXCL8 was cleaved by MMP-9 or MMP-13 (the smaller sized band is indicated by a black arrowhead). In contrast, just one band (arrow) was obtained after recombinant CCL2 was cleaved by MMP-9 or MMP-13.

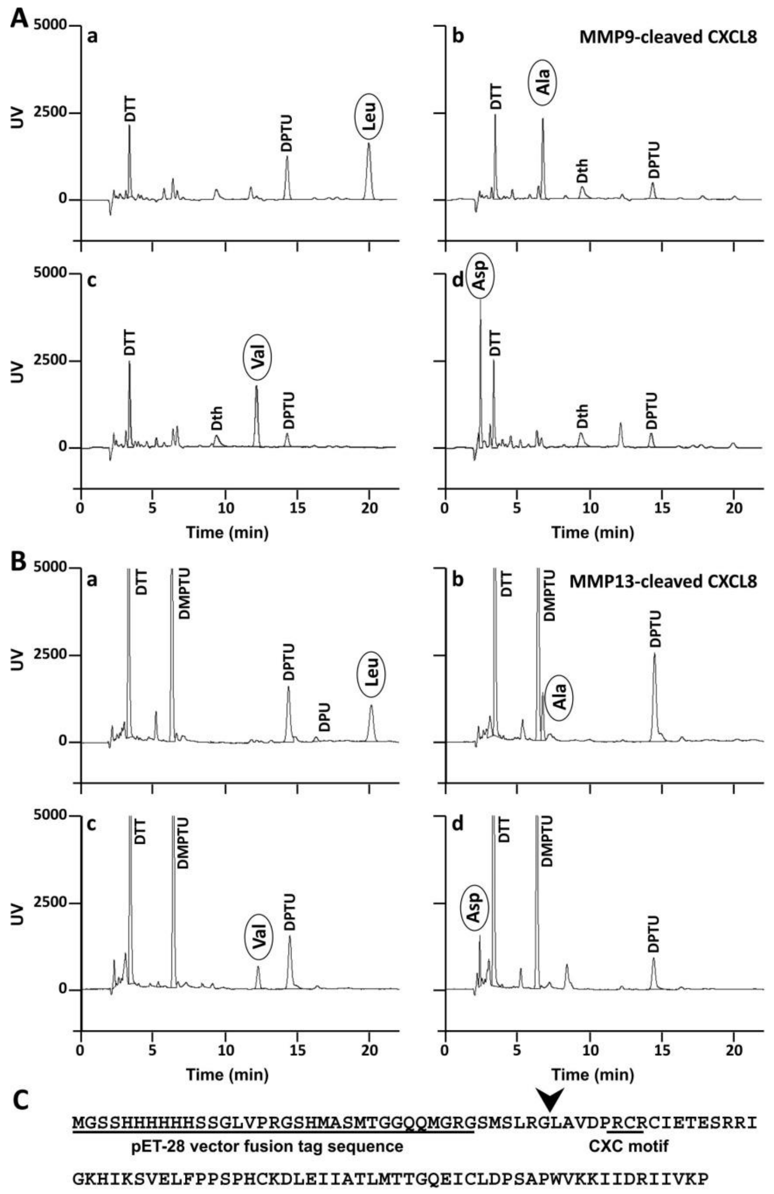

Figure S4. N-terminal sequencing of MMP9- and MMP13-cleaved CXCL8. To determine the cutting sites on CXCL8, N-terminal sequencing was conducted on the smaller of the two (A) MMP9-or (B) MMP13-cleaved CXCL8 fragments (see black arrowhead in Figs 7A and S3) using the Edman degradation method. The amino acids bounded by the ellipses show the first 4 amino acids of the N-terminus of the cleaved form of CXCL8. (C) The whole amino acid sequence of recombinant CXCL8. The vector fusion tag and CXC motif are underlined and the MMP9/MMP13 cleaving site is shown by a black arrowhead.

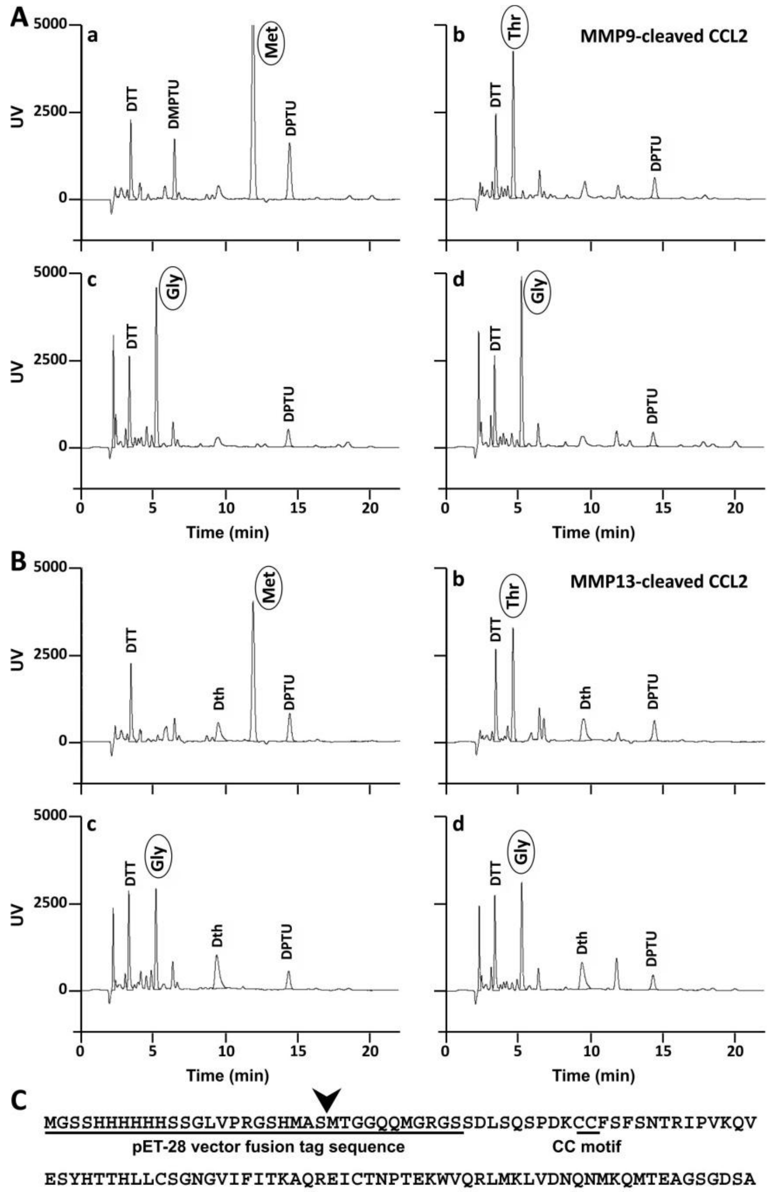

Figure S5. N-terminal sequencing of MMP9- and MMP13-cleaved CCL2. To determine the cutting sites on CCL2, N-terminal sequencing was conducted on the (A) MMP9-or (B) MMP13-cleaved CCL2 fragments (see black arrow in Figs 7A and S3) using the Edman degradation method. The amino acids bounded by the ellipses show the first 4 amino acids of the N-terminus of the cleaved form of CCL2. (C) The whole amino acid sequence of recombinant CCL2. The vector fusion tag and CC motif are underlined and the MMP9/MMP13 cleaving site is shown by a black arrowhead.

Table S1 The numbers of neutrophils and macrophages in the injured area

Time point	Intact		0.5 dpc		1 dpc		4 dpc		7 dpc		14 dpc	
Cell No. ($\times10^3$) per mm^3	Control	GM6001	Control	GM6001	Control	GM6001	Control	GM6001	Control	GM6001	Control	GM6001
Neutrophils	0.53	0.00	69.75	3.67	97.73	31.82	30.02	14.14	13.74	15.07	15.18	11.97
Macrophages	1.58	1.08	6.41	0.96	21.21	7.37	12.02	3.31	2.77	3.04	3.40	2.78
